# Supplementary material for: Genome Analysis of Methicillin-Resistant and Methicillin-Susceptible Staphylococcus aureus ST398 Strains Isolated from Patients with Invasive Infection
Source: Microorganisms. 2023 May 30;11(6):1446. doi: 10.3390/microorganisms11061446 (PMC10302279; doi:10.3390/microorganisms11061446)
Supplement: Supplementary file 1 [file microorganisms-11-01446-s001.zip › microorganisms-2407801-supplementary.pdf]

Supplementary Materials

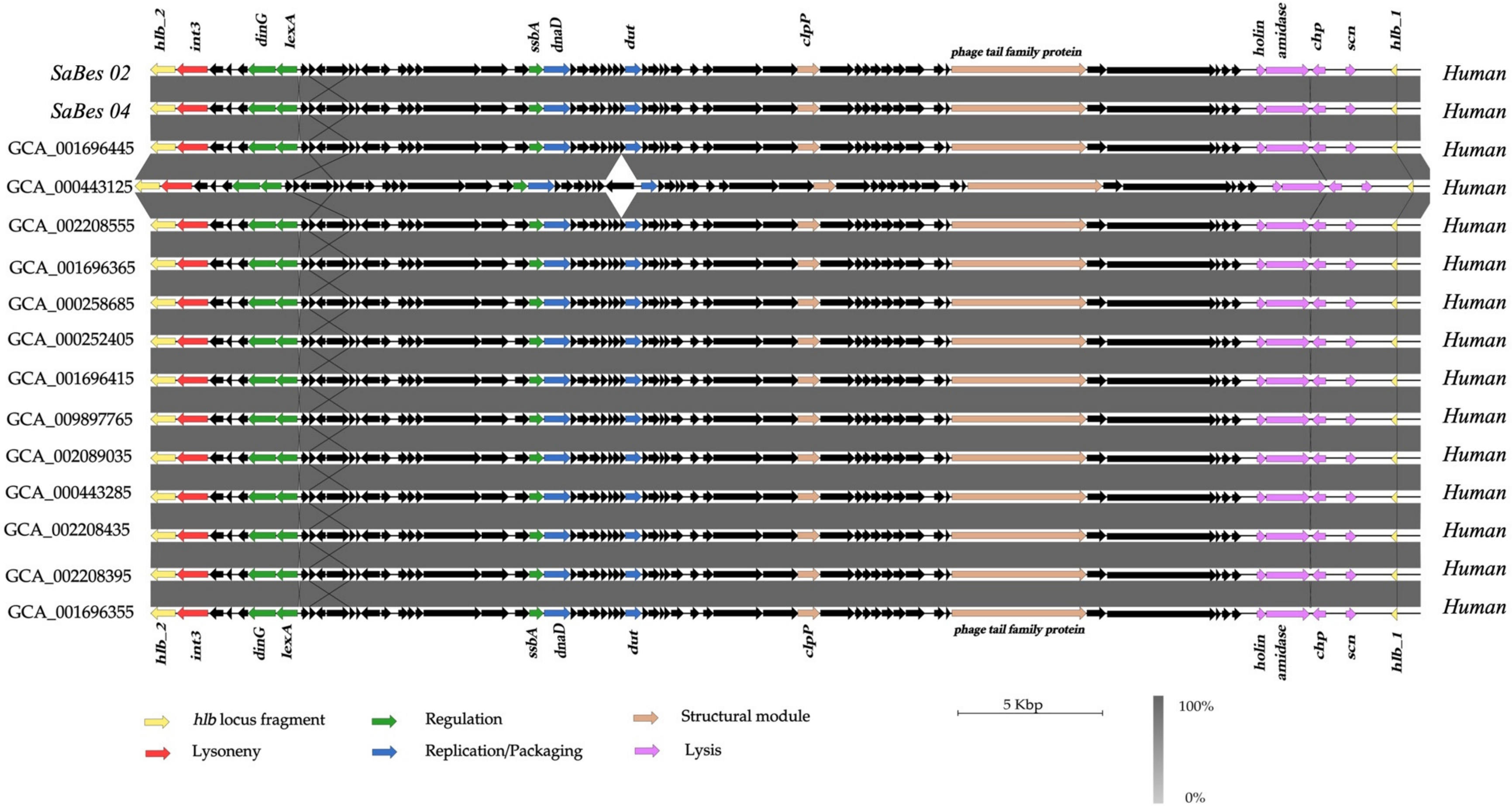

Figure S1. Comparison of the φSa3 prophage genomes of two MSSA isolates (current study) and human MSSA isolates from NCBI.

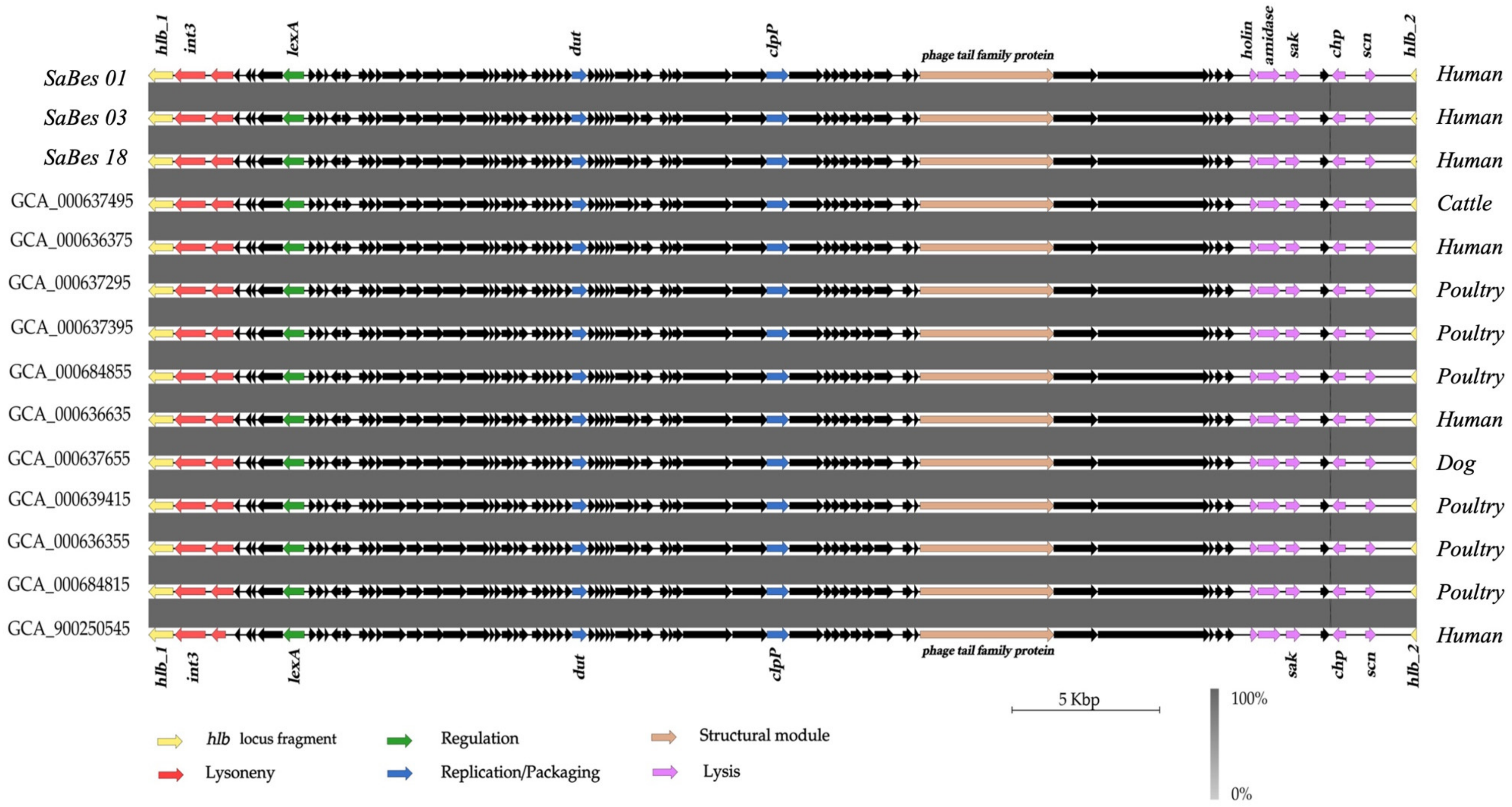

Figure S2. Comparison of the φSa3 prophage genomes of 3 MRSA (current study) and MRSA isolates from NCBI.
